# A rapid progressive and fatal case of Non-Hodgkin’s lymphoma in a newly diagnosed HIV patient

**DOI:** 10.1186/1471-2334-14-S7-P3

**Published:** 2014-10-15

**Authors:** Liana-Cătălina Gavriliu, Elisabeta-Otilia Benea, Șerban Benea, Roxana Dumitriu, Georgeta Ducu, Daniela Camburu, Alina Cozma, Manuela Podani, Mihaela Ionică, Cosmina Andrei, Mădălina Simoiu, Nicoleta Bunescu

**Affiliations:** 1 National Institute for Infectious Diseases "Prof. Dr. Matei Balş", Bucharest, Romania; 2 Carol Davila University of Medicine and Pharmacy, Bucharest, Romania; 3 Emergency Clinical Hospital “Sf. Pantelimon”, Bucharest, Romania

## Background

Non-Hodgkin's lymphoma (NHL) is one of the frequent and severe oncologic pathologies associated with HIV infection. Unlike in immunocompetent patients, this pathology manifests more aggressively in HIV-positive patients, frequently with severe systemic extranodal involvement, affecting the gastrointestinal tract, liver, bone marrow, central nervous system (CNS). Along extranodal involvement, the low level of CD4 at diagnosis, previous history of AIDS-defining illness, the NHL advanced stage, high LDH level and advanced age, are unfavorable prognostic factors for NHL associated with HIV infection.

## Case report

We present the case of a 27 year-old patient, admitted to INBI "Prof. Dr. Matei Balş" with axillary tumoral mass with rapid growth in size within past two months, back pain that irradiated towards the lower limbs, abdominal pain, diplopia. The patient was initially hospitalized in a surgical oncology clinic where a biopsy from the tumoral mass was performed and the pathological aspect revealed granulomatous lymphadenitis. In our clinic we confirmed the presence of HIV infection, with a value of CD4 T lymphocytes of 442/cmm. On the 6^th^ day of hospitalization, the patient developed fever. Because of the previous histopathology exam and before we elucidated the diagnosis, different causes of febrile syndrome and enlarged lymph nodes were taken into account: tuberculosis, fungal infection, for which the appropriate therapy was instituted. At the same time, during the two weeks of admission we performed lymph node and bone marrow biopsy that confirmed the diagnosis of NHL stage IV with middle B-cell. Imaging studies (brain MRI, chest CT, abdominal ultrasound) certified the presence of brain, liver, intrathoracic and paravertebral involvement. The patient was promptly started on HAART and he was referred to the hematology clinic for NHL treatment. At 24 hours after the transfer, the patient died.

## Conclusion

Our case illustrates the evolution of an aggressive NHL in a young patient, recently diagnosed with HIV infection, with a good immune status, which is inconsistent with classical prognostic factors. In addition, due to multisystem involvement of NHL frequently encountered in these patients, the differential diagnosis can be extensive and a positive diagnosis represents an emergency and is essentially established through histopathology. This involves a close and effective interdisciplinary collaboration.

## Consent

Written informed consent was obtained from the patient's next of kin for publication of this Case report and any accompanying images. A copy of the written consent is available for review by the Editor of this journal.

